# *Salmonella enterica* as a Complementary Model to LPS for Immune Stress in Weaned Piglets: Systemic and Intestinal Alterations

**DOI:** 10.3390/ani16020311

**Published:** 2026-01-20

**Authors:** Li Dong, Zhiyan Liu, Wenxi Li, Changwei Zhang, Haoyang Yuan, Jun Liu, Hongrong Wang, Lihuai Yu

**Affiliations:** 1 Department of Animal Science and Technology, College of Animal Science and Technology, Yangzhou University, No. 48 of East Wenhui Road, Yangzhou 225009, China; donglijiayou@126.com (L.D.); mz120241680@stu.yzu.edu.cn (Z.L.); 241902310@stu.yzu.edu.cn (W.L.); zweijie2021@163.com (C.Z.); yhy980603@163.com (H.Y.); shiqin1214@126.com (J.L.); hrwang@yzu.edu.cn (H.W.); 2 Institute of Animal Nutrition, Sichuan Agricultural University, Chengdu 611130, China

**Keywords:** *Salmonella*, immune stress, weaned piglets, lipopolysaccharide, intestine, cytokines, T lymphocytes

## Abstract

Lipopolysaccharide (LPS) is widely used to model immune stress in weaned piglets, but it does not fully replicate the pathophysiological alterations induced by live bacterial infection. This study therefore established an oral *Salmonella enterica* (*SE*) challenge model and systematically compared its effects with those of LPS to evaluate its potential as a complementary immune stress paradigm. Forty piglets were assigned to five groups: control (saline), LPS (intraperitoneal, 100 μg/kg BW), and three SE groups receiving low-, middle-, or high-dose oral SE (1 × 10^8^ CFU/mL, 2 × 10^8^ CFU/mL, or 3 × 10^8^ CFU/mL in a 10 mL saline volume, respectively). Both LPS and SE significantly reduced average daily gain, while only SE challenge decreased colon length. A transient rectal temperature elevation occurred at 8 h in all challenged groups, persisting at 12 h in the LPS and high-dose SE groups. Serum cytokine analysis revealed that LPS induced early but transient interleukin-12 and tumor necrosis factor-α elevation at 8 h, followed by sustained suppression of interferon-γ, interleukin-6, and interleukin-8. In contrast, the middle-dose SE triggered robust increases in multiple pro-inflammatory cytokines at 24 h. Both challenges significantly reduced the CD4^+^/CD8^+^ T cell ratios in blood and lymphoid organs and decreased intestinal interleukin-10 levels. SE infection produced more severe intestinal pathology, including dose-dependent villus perforations, microvillus disorganization, and mitochondrial cristae vacuolization, beyond the villus shortening and goblet cell reduction observed in both groups. While both LPS and SE induced immune stress and intestinal injury, SE infection caused more severe and comprehensive pathophysiological alterations. Oral administration of 2 × 10^9^ CFU SE for 24 h established a physiologically relevant immune stress model that effectively mimics natural *Salmonella* infection in weaned piglets, providing a valuable tool for studying enteric diseases and evaluating interventions.

## Simple Summary

The post-weaning period is a critical transition phase in pig production, during which piglets exhibit increased susceptibility to enteric pathogens and associated intestinal disorders. To investigate the underlying mechanisms, lipopolysaccharide (LPS), a component of bacterial cell walls, is frequently used to induce immune stress in experimental models. However, LPS administration alone fails to recapitulate the full spectrum of pathological changes caused by live bacterial infections. In this study, we established an oral infection model using *Salmonella enterica* (SE), a major enteric pathogen, and systematically compared its effects with those of LPS in weaned piglets. Our results demonstrated that while both challenges in duced immune activation and intestinal injury, SE infection provoked more severe and sustained systemic inflammation, along with distinctive gut structural damage, such as villus perforations and mitochondrial damage, that were not observed in the LPS group. Notably, an oral dose of 2 × 10^9^ CFU SE for 24 h established a reproducible and physiologically relevant model that closely mimics natural *Salmonella* infection. This optimized model provides a valuable experimental platform for further research on intestinal diseases and for evaluating nutritional or therapeutic interventions aimed at enhancing gut health in swine, with potential benefits for animal welfare and sustainable pork production.

## 1. Introduction

Post-weaning stress represents a critical period in swine production, significantly increasing piglet susceptibility to enteric pathogens and associated immune dysregulation [1]. To study the ensuing pathophysiology, immune stress modeling in weaned piglets has primarily relied on lipopolysaccharide (LPS) challenge or live *Escherichia coli* infection. LPS, a key component of Gram-negative bacterial cell walls, is extensively utilized to induce acute systemic inflammation [2,3]. However, its model fidelity is limited, as it typically elicits transient systemic inflammation without reproducing the characteristic intestinal pathology of a live bacterial infection [4]. Conversely, while *E. coli* is also a common enteric pathogen, it often fails to provoke a robust and consistent immune activation in challenge models, limiting its utility for immune stress studies [5]. These constraints highlight a need for complementary models that better recapitulate the complex host responses to enteric pathogens, particularly in the intestinal compartment.

*Salmonella enterica* (SE) presents a promising complementary model, being a primary enteric pathogen capable of causing severe gastroenteritis in both animals and humans [6]. Its potential for modeling is underpinned by findings that SE challenge can induce significant pyrexia and marked alterations in serum immune markers [7,8]. Furthermore, the specific SE strain 50336 used in this study has demonstrated a pronounced ability to infect porcine jejunal epithelial cells (IPEC-J2) in vitro, indicating a high affinity for the porcine intestinal tract [9]. These findings suggest that oral SE challenge could effectively mimic key aspects of natural infection. More importantly, SE possesses virulence mechanisms, such as type III secretion systems (T3SS), that are absent in purified LPS and are known to drive more profound gut morphological damage, including villus perforations and disruption of tight junctions [10]. Consequently, an SE-based model may induce more severe and comprehensive intestinal pathophysiological alterations, providing a complementary approach to the classic LPS model, especially for studies focused on gut-specific immune responses and structural damage.

Therefore, this study aimed to directly address the limitations of existing models by systematically comparing the pathophysiological differences between the standard LPS challenge and an optimized oral SE infection in weaned piglets. We focused on a multidimensional assessment of systemic inflammation, intestinal immunity, and gut morphology to evaluate SE as a complementary model for immune stress in weaned piglets.

## 2. Materials and Methods

### 2.1. Bacterial Strain

The *Salmonella enterica* strain 50336 was provided by Professor Guoqiang Zhu (College of Veterinary Medicine, Yangzhou University).

### 2.2. Animals and Experimental Design

Forty piglets weaned at 21 days of age (28 days old at study initiation) were randomly allocated to five groups (n = 8/group): CTR (control), orally administered 10 mL of 0.9% saline; LPS, intraperitoneally injected with 100 μg/kg body weight LPS (*E. coli* O55:B5; Sigma L6529, Sigma-Aldrich, St. Louis, MO, USA); LSE, orally administered 1 × 10^8^ CFU/mL SE in 10 mL saline; MSE, orally administered 2 × 10^8^ CFU/mL SE in 10 mL saline; HSE, orally administered 3 × 10^8^ CFU/mL *SE* in 10 mL saline. The commercially available *E. coli* O55:B5 LPS was selected as it represents a standardized and widely referenced agent for inducing endotoxemia in swine, facilitating direct comparison with established models [4,11]. The LPS dose followed established protocols [4,11]. Piglets received a basal diet (Supplementary Materials Table S1; Yangzhou University Feed Factory) free of antibiotics, formulated according to the National Research Council (2012). The piglets were obtained from a commercial farm with a standard health management protocol but were not subjected to any vaccination program against *Salmonella* or other enteric pathogens prior to or during the trial. At 8, 12, 24, and 36 h post-challenge, rectal temperature was recorded and venous blood was collected. Six piglets per group were euthanized at 36 h for tissue sampling. The selected time points (8, 12, 24, and 36 h post-challenge) were based on the established kinetics of immune and pathophysiological responses in weaned piglet models [12,13,14].

### 2.3. Sample Collection

Blood samples were collected from the anterior vena cava using a sterile 21-gauge needle attached to a 5 mL syringe. The venipuncture site was disinfected with 70% ethanol prior to collection. Samples were collected at for cytokine analysis (IFN-γ, IL-6, IL-8, IL-12, TNF-α) and immune cell profiling. At 36 h, the abdominal cavity was opened, the intestine was separated, and the length of the duodenum, jejunum and ileum were measured. A 2-cm segment from the mid-section of the small intestine was rinsed with PBS, longitudinally incised, treated with 0.25% trypsin to remove mucus, and fixed in 2.5% glutaraldehyde for scanning and transmission electron microscopy. Segments (2 cm) from the mid-jejunum and mid-ileum were fixed in 4% formaldehyde for histological study. The intestinal contents of jejunum and ileum were flushed with pre-cooled PBS, the moisture was dried with filter paper, and the mucosa was gently scraped with a slide. The samples were then flash-frozen in liquid nitrogen for cytokine analysis. Fresh samples of the thymus, spleen, and mesenteric lymph nodes were collected immediately for T Lymphocyte subset analysis.

### 2.4. Cytokine Quantification

Serum and intestinal cytokine levels (IL-1β, IL-6, IL-8, IL-10, IL-12, TNF-α, IFN-γ) were measured using commercial ELISA kits (Nanjing Jiancheng Bioengineering Institute, Nanjing, China; Cat# H002, H008, H009, H010, H025, H052) according to the manufacturer’s protocols.

### 2.5. T Lymphocyte Subset Analysis

Heparinized whole blood (100 μL) was incubated with FITC-conjugated anti-CD4 (Solarbio K009445M; Solarbio Science & Technology Co., Ltd., Beijing, China) and PE-conjugated anti-CD8α (Solarbio K009446M; Solarbio Science & Technology Co., Ltd., Beijing, China; 10 μL each) for 30 min at 4 °C in dark. Erythrocytes were lysed with 3 mL of lysis buffer, samples were centrifuged (500× *g*, 5 min), and pellets were resuspended in 1% paraformaldehyde for flow cytometry [15]. Homogenates of thymus, spleen, and mesenteric lymph nodes were filtered through a 300-μm nylon mesh), centrifuged (100× *g*, 8 min, 4 °C), washed twice with PBS, and stained identically to blood samples.

### 2.6. Electron Microscopy

For scanning electron microscope (SEM): Fixed tissues were washed with 0.1 M PBS, post-fixed in 1% OsO_4_, dehydrated in a graded ethanol series (30–100%), critical-point dried, sputter-coated with gold, and imaged (ZEISS GeminiSEM 300; Carl Zeiss AG, Oberkochen, Germany) [16]. For transmission electron microscopy (TEM): Samples were fixed in 1% OsO_4_ (2 h, dark), dehydrated in ethanol/acetone, embedded in resin, polymerized (37 °C overnight; 60 °C, 48 h), ultrathin-sectioned, stained, and imaged (Hitachi HT7800; Hitachi High-Tech Corporation, Tokyo, Japan) [17].

### 2.7. Histological Analysis

Formaldehyde-fixed tissues were paraffin-embedded, sectioned (5 μm), and stained with hematoxylin and eosin (Solarbio G1120; Solarbio Science & Technology Co., Ltd., Beijing, China). Villus height, crypt depth, and villus height-to-crypt depth ratio (V/C) were quantified using Image-Pro Plus 6.0 (Media Cybernetics, Rockville, MD, USA) under an Olympus BX51 microscope (Olympus Corporation, Tokyo, Japan) [18].

### 2.8. Goblet Cell Staining

Goblet cells were stained with Alcian Blue (Solarbio G1560; Solarbio Science & Technology Co., Ltd., Beijing, China) according to the manufacturer’s instructions. Five intact villi per section were imaged (Nikon Eclipse E100, Nikon Instruments Inc., Tokyo, Japan), and goblet cells were quantified using Image J software 17.0.

### 2.9. Statistical Analysis

All data were analyzed using SPSS 25.0 (IBM Corp., Armonk, NY, USA). Group differences were assessed by one-way analysis of variance (ANOVA) followed by Duncan’s post hoc test for multiple comparisons. Figures were generated using GraphPad Prism 8.0.2 (GraphPad Software, San Diego, CA, USA). Statistical significance was established at *p* < 0.05.

## 3. Results

### 3.1. Body Weight, Colon Length and Rectal Temperature

**Figure 1:**
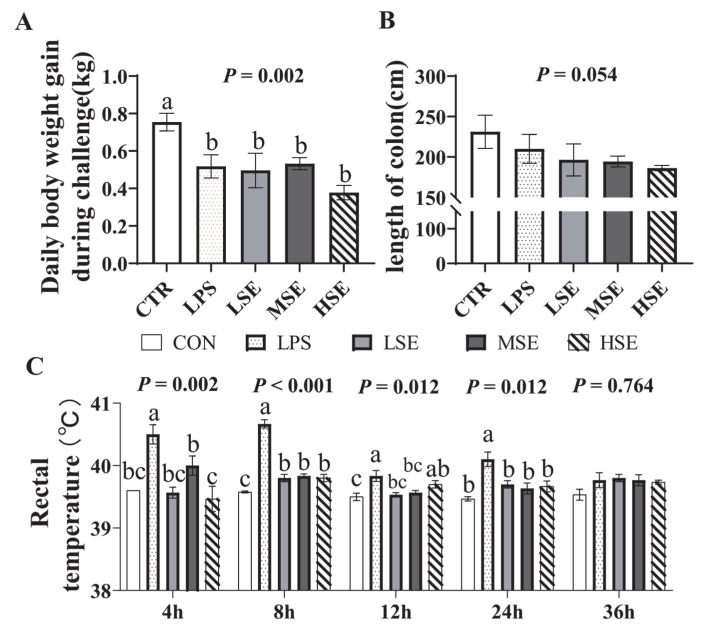
Body Weight, Colon Length and Rectal Temperature. Average daily gain (ADG) of piglets in the control (CTR), lipopolysaccharide (LPS), and low-, medium-, and high-dose *Salmonella enterica* (LSE, MSE, HSE)-challenged groups (**A**). Colon length of piglets from each treatment group (**B**). Rectal temperature measured at 0, 4, 8, 12, 24, and 36 h post-challenge (**C**). Different letters (a, b, c) above bars indicate statistically significant differences between groups (*p* < 0.05). Values are presented as mean ± SEM (n = 6).

Compared to the CTR group, the average daily weight gain (ADG) was significantly reduced in all challenged groups (LPS, LSE, MSE, HSE; *p* = 0.002; Figure 1A). Colon length tended to be shorter in SE-challenged groups compared to the CTR group (*p* = 0.054; Figure 1B). Rectal temperature increased significantly at 8 h post-challenge in the LPS and all SE groups compared to CTR (*p* < 0.001). By 12 h, significantly elevated temperatures persisted in the LPS and HSE groups (*p* = 0.012; Figure 1C).

### 3.2. Levels of Cytokines in Serum and Small Intestine

**Figure 2:**
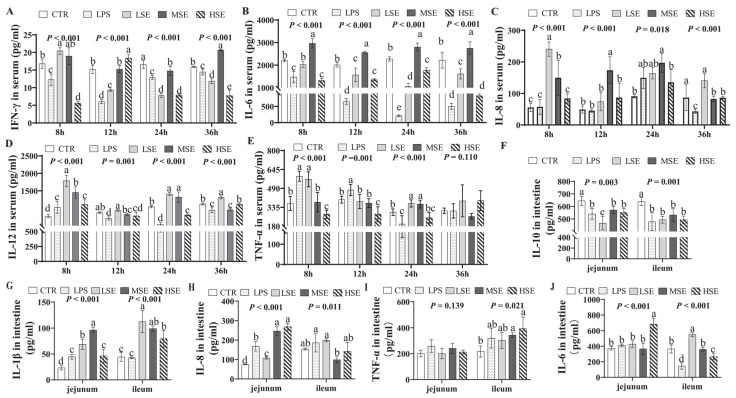
Cytokine profiles in serum and intestinal tissues. Serum levels of IFN-γ (**A**), IL-6 (**B**), IL-8 (**C**), IL-12 (**D**), and TNF-α (**E**) of piglets in the control (CTR), lipopolysaccharide (LPS), and low-, medium-, and high-dose *Salmonella enterica* (LSE, MSE, HSE)-challenged groups were measured at 8, 12, 24, and 36 h post-challenge. At 36 h post-challenge, the levels of IL-10 (**F**), IL-1β (**G**), IL-8 (**H**), TNF-α (**I**), and IL-6 (**J**) were quantified in the jejunum and ileum. Different letters (a, b, c, d) above bars indicate statistically significant differences between groups (*p* < 0.05). Values are presented as mean ± SEM (n = 6).

At 12 h post-stimulation, serum IFN-γ levels were significantly elevated in the HSE group (*p* < 0.05), while MSE-induced significant increases in serum IL-6 at 8, 12, 24, and 36 h (*p* < 0.05) (Figure 2A,B). Serum IL-8 levels were significantly higher in the LSE and MSE groups at 8 h, with sustained elevations in the MSE group at 12 and 24 h (*p* < 0.05) (Figure 2C). At 8 h, both LPS and SE groups showed significantly increased serum IL-12, and the LPS and LSE groups exhibited significantly higher serum TNF-α (*p* < 0.05) (Figure 2D,E). Conversely, the LPS group had significantly reduced serum IFN-γ and IL-6 at all time points, along with significantly lower IL-8 at 36 h (*p* < 0.05) (Figure 2A–C).

At 36 h post-challenge, jejunal and ileal IL-10 levels were significantly reduced in both LPS and SE groups relative to the CTR group (*p* < 0.05) (Figure 2F). In contrast, jejunal IL-1β was elevated in the LPS group, and both jejunal and ileal IL-1β were significantly increased in SE groups (*p* < 0.05) (Figure 2G). Jejunal IL-8 levels were significantly higher in the LPS, MSE, and HSE groups compared to CTR (*p* < 0.05) (Figure 2H). Additionally, the HSE group showed significantly increased ileal TNF-α and jejunal IL-6, while the LSE group had elevated ileal IL-6 (*p* < 0.05 vs. CTR) (Figure 2I,J).

### 3.3. T-Lymphocyte Subsets in Blood and Immune Organs

**Figure 3:**
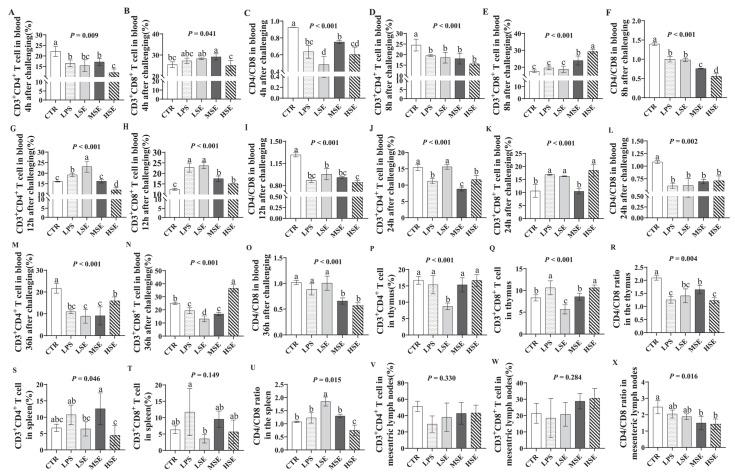
CD4^+^/CD8^+^ T lymphocyte ratio in peripheral blood and immune organs. The ratio of CD4^+^ to CD8^+^ T lymphocytes was analyzed in blood samples collected at (**A**–**C**) 4 h, (**D**–**F**) 8 h, (**G**–**I**) 12 h, (**J**–**L**) 24 h, and (**M**–**O**) 36 h post-challenge from piglets in the control (CTR), lipopolysaccharide (LPS), and low-, medium-, and high-dose Salmonella enterica (LSE, MSE, HSE)-challenged groups. CD4^+^/CD8^+^ T lymphocyte ratio in the thymus (**P**–**R**), spleen (**S**–**U**) and mesenteric lymph nodes (**V**–**X**) at 36 h. Different letters (a, b, c, d) above bars indicate statistically significant differences between groups (*p* < 0.05). Values are presented as mean ± SEM (n = 6).

In blood, the CD4^+^/CD8^+^ T lymphocyte ratio was significantly decreased in the LPS and SE groups at 4, 8, 12, 24, and 36 h post-challenge (*p* < 0.05) (Figure 3A–O). At 36 h, this ratio was also significantly lower in the thymus, spleen, and mesenteric lymph nodes of LPS- and SE-challenged piglets compared to the control group (*p* < 0.05) (Figure 3P–X).

### 3.4. Intestinal Morphology

**Figure 4:**
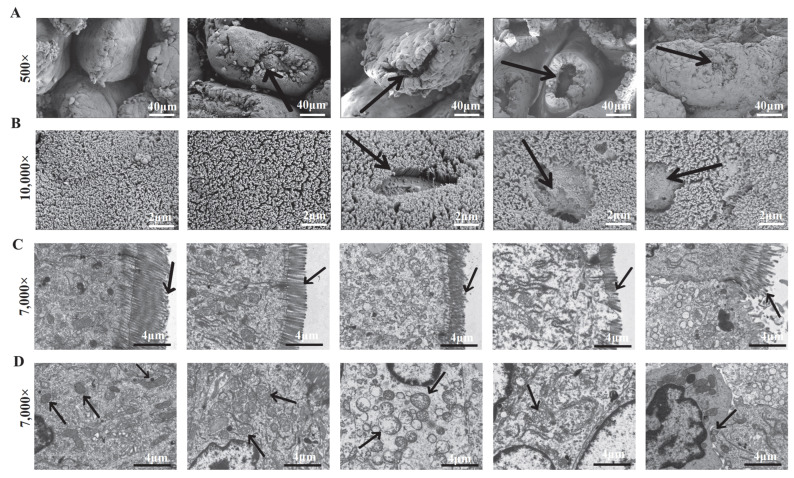
Ultrastructural alterations in the small intestinal epithelium revealed by scanning electron microscopy (SEM) and transmission electron microscopy (TEM). SEM images showing damage to villus tips in the small intestine of challenged piglets (representative images shown) (**A**). SEM images depicting surface holes (arrows) specific to the enterocytes of SE-challenged piglets (**B**). TEM micrographs showing shortened and disorganized microvilli (MV) and mitochondrial swelling (arrows) in challenged groups (**C**). High-magnification TEM images revealing severe mitochondrial damage, including vacuolated cristae (arrowheads), in the SE-challenged groups (**D**). Values are presented as mean ± SEM (n = 6).

**Figure 5:**
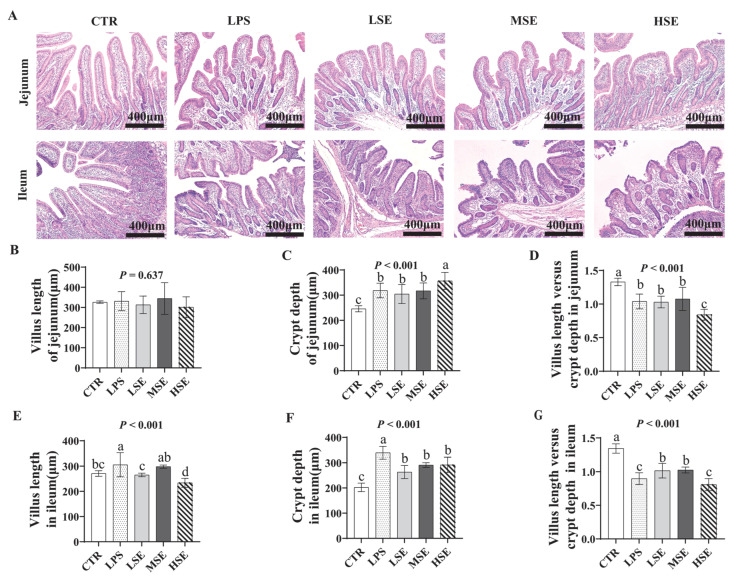
Histological analysis of jejunal and ileal morphology. Representative hematoxylin-and-eosin (H&E)-stained sections of the jejunum and ileum of piglets in the control (CTR), lipopolysaccharide (LPS), and low-, medium-, and high-dose *Salmonella enterica* (LSE, MSE, HSE)-challenged groups (**A**). The villus height, crypt depth and villus height to crypt depth (V/C) ratio of the jejunum (**B**–**D**) and ileum (**E**–**G**). Different letters (a, b, c, d) above bars indicate statistically significant differences between groups (*p* < 0.05). Values are presented as mean ± SEM (n = 6).

Scanning electron microscopy revealed villus tip damage in the small intestine of LPS and SE groups (Figure 4A), with surface perforations specific to SE-challenged piglets (Figure 4B). Microvilli were shorter in LPS and SE groups than in CTR, and SE groups exhibited disorganized microvilli in a dose-dependent manner (Figure 4C). Mitochondrial swelling was observed in both LPS and SE groups, with more severe damage in SE groups, including vacuolated cristae (Figure 4C,D). Hematoxylin–eosin (HE) staining showed significantly reduced villus height/crypt depth (V/C) ratios in the jejunum and ileum of LPS and *SE* groups compared to CTR (*p* < 0.05) (Figure 5).

### 3.5. Number of Goblet Cells

**Figure 6:**
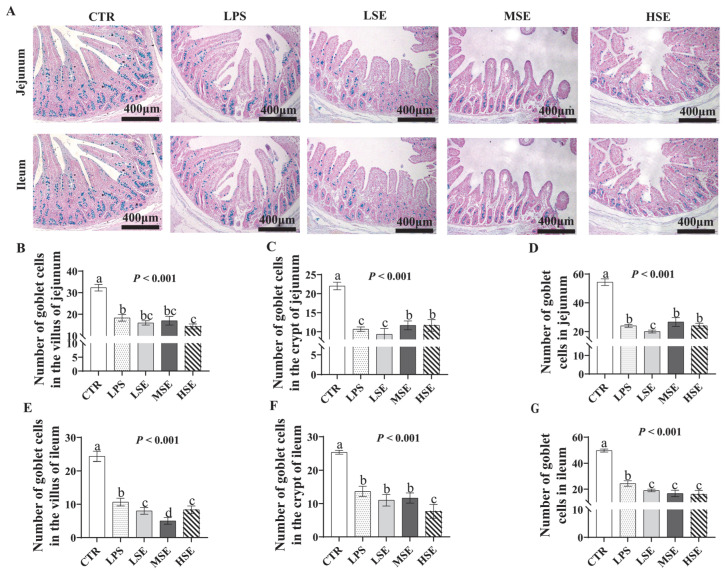
Goblet cell count in the jejunum. Representative histochemical images (Alcian Blue staining) of jejunal sections showing goblet cells in villi and crypts of jejunum and ileum of piglets in the control (CTR), lipopolysaccharide (LPS), and low-, medium-, and high-dose *Salmonella enterica* (LSE, MSE, HSE)-challenged groups (**A**). Quantification of goblet cells in the villi and crypts of jejunum (**B**–**D**) and ileum (**E**–**G**). Different letters (a, b, c, d) above bars indicate statistically significant differences between groups (*p* < 0.05). Values are presented as mean ± SEM (n = 6).

In the jejunum and ileum, the number of goblet cells in villi and crypts was significantly lower in LPS and SE groups than in CTR (*p* < 0.05) (Figure 6).

## 4. Discussion

Lipopolysaccharide (LPS) remains a cornerstone for modeling intestinal inflammatory injury, particularly in simulating Gram-negative bacterial infections such as *Salmonella enterica* (SE) [2,3]. However, our findings demonstrate that the immunological and pathophysiological distinctions between purified endotoxin exposure and live bacterial invasion are substantial, especially in the intestinal compartment. We systematically evaluated these responses in weaned piglets and demonstrated that oral SE infection (optimally at 2 × 10^9^ CFU) offers a complementary model. This model captures essential features of natural gut infection that LPS alone does not fully represent. The results of this study supported the utility of the SE model as a valuable adjunct to existing paradigms, particularly for studies focusing on intestinal pathology and sustained local immune activation.

The observed suppression of average daily gain (ADG) in both LPS- and SE-challenged piglets reflects the metabolic burden of systemic inflammation. This growth impairment likely stems from impaired nutrient absorption due to villus shortening and mitochondrial dysfunction [19], combined with pro-inflammatory cytokine-mediated anorexia [20]. Notably, SE infection induced more severe growth retardation and a unique reduction in colon length, aligning more closely with clinical reports of *Salmonella*-associated enteropathy characterized by persistent diarrhea and malabsorption [21]. The rectal temperature profiles further distinguished these stressors. LPS triggered a rapid, sharp hyperthermia (>40.5 °C at 8 h), characteristic of acute endotoxin shock mediated primarily via TLR4/MyD88-dependent signaling [22]. In contrast, SE elicited a more dose-dependent and sustained pyrexia, underscoring its engagement of multiple pattern recognition receptors (PRRs), including NLRs and cytosolic sensors, a hallmark of live bacterial invasion [23].

The cytokine responses unveiled a fundamental divergence in immune activation kinetics. The response to LPS was biphasic: an early surge in TNF-α and IL-12 was followed by the sustained suppression of IFN-γ, IL-6, and IL-8. This pattern is a classic manifestation of endotoxin tolerance, a compensatory mechanism that prevents uncontrolled inflammation [24]. This pattern starkly contrasts with the sustained, robust elevation of multiple pro-inflammatory cytokines, particularly IL-6 and IL-8, observed at 24 h post-SE infection, especially in the MSE group. This sustained response indicates persistent bacterial antigen presentation and inflammasome activation [25], more accurately mimicking the ongoing immune battle during a natural infection. Critically, the 2 × 10^9^ CFU SE dose generated optimal and reproducible cytokine kinetics for modeling. The spatial heterogeneity in intestinal cytokines, with ileal IL-6 dominating in low-dose *SE* and jejunal IL-8 prevailing at higher doses, may reflect differential microbial density or epithelial Toll-like receptor expression along the intestinal tract [26]. The concurrent suppression of the anti-inflammatory cytokine IL-10 in both jejunum and ileum across challenged groups underscores a critical disruption of the anti-inflammatory feedback loop, likely permitting uncontrolled neutrophil infiltration via IL-8-mediated chemotaxis [27].

A key finding was the significantly reduced CD4^+^/CD8^+^ T cell ratios across peripheral blood and all examined lymphoid organs (thymus, spleen, mesenteric lymph nodes). This signifies a profound, systemic immunosuppression induced by both challenges. Mechanistically, TNF-α overexpression can promote CD4^+^ T cell apoptosis [28], while chemokine gradients may sequester CD8^+^ T cells at inflamed intestinal sites [29]. The observed thymic involvement suggests a potential disruption in T cell ontogeny [30]. Moreover, dysfunction in the mesenteric lymph nodes, which are critical for intestinal immune surveillance, would severely compromise local immune responses [31]. This multi-organ immune impairment heightens susceptibility to secondary infections, a finding with critical implications for swine health management.

While both LPS and SE induced common morphological injuries like villus blunting, reduced V/C ratio, and microvillus atrophy, SE infection uniquely caused severe ultrastructural lesions. The dose-dependent villus perforations and mitochondrial cristae vacuolization observed specifically in SE-challenged piglets indicated direct epithelial cytotoxicity that extends beyond the effects of purified endotoxin. These distinctive pathological features are consistent with the action of *Salmonella*’s type III secretion systems (T3SS), which are known to disrupt tight junctions by redistributing claudin-1 and occludin [32]. The observed depletion of goblet cells across challenged groups further compromises the mucus barrier, but this effect was amplified in a dose-dependent manner by SE infection, facilitating potential pathogen translocation.

Collectively, our data robustly establish that oral challenge with 2 × 10^9^ CFU SE at 24 h post-infection constitutes a physiologically superior model for immune stress in weaned piglets. This model elicits severe and sustained systemic and intestinal immune activation, induces characteristic morphological damage in the gut, and leads to measurable growth suppression, thereby closely mimicking key features of natural infection. The resulting growth impairment and intestinal pathology translate directly into economic losses in swine production through reduced feed efficiency and increased morbidity. The optimized SE challenge model presented here offers a valuable platform for screening nutritional and therapeutic interventions aimed at mitigating such losses, ultimately supporting strategies to enhance gut health and improve productivity. It should be noted that while the sample size (n = 6 per group) followed established standards in porcine challenge models and was sufficient to detect significant differences in primary outcomes [33,34], a formal a priori power calculation was not conducted. Future studies applying this model, especially when evaluating interventions with subtler effects, would benefit from such calculations to strengthen experimental design. Finally, this study focused on acute responses within 36 h; longer-term observations would help clarify recovery dynamics and adaptive immunity. The model also remains to be tested with different *Salmonella* strains. Moving forward, this platform is well-suited for assessing feed additives, vaccines, and probiotics aimed at improving gut health. Deeper mechanistic insights could be gained by integrating transcriptomic or metabolomic approaches, and by evaluating specific interventions such as those targeting IL-10 pathway restoration [35] or mitochondrial integrity protection [36], with the goal of reducing post-weaning gastrointestinal morbidity in commercial swine production.

## 5. Conclusions

In summary, this study demonstrates that while both LPS challenge and *Salmonella enterica* (SE) infection induced immune stress in weaned piglets, oral SE infection provoked a more severe and comprehensive pathophysiological response. SE infection resulted in more pronounced growth impairment, sustained systemic inflammation, and profound intestinal damage, including distinctive lesions such as villus perforations and mitochondrial vacuolization not observed in the LPS model. Crucially, an oral dose of 2 × 10^9^ CFU SE for 24 h was optimized to establish a robust and physiologically relevant immune stress model. This validated protocol provides a potential tool for future research on intestinal immunology and for evaluating nutritional and therapeutic interventions in weaned piglets.

## Data Availability

The data of the study are available from the corresponding authors upon reasonable request.

## Supplementary Materials

The following supporting information can be downloaded at: https://www.mdpi.com/article/10.3390/ani16020311/s1, Table S1: Composition and nutrient levels of basal diets (air-dry basis).
