# Supplementary material for: Salmonella enterica as a Complementary Model to LPS for Immune Stress in Weaned Piglets: Systemic and Intestinal Alterations
Source: Animals (Basel). 2026 Jan 20;16(2):311. doi: 10.3390/ani16020311 (PMC12837481; doi:10.3390/ani16020311)
Supplement: Supplementary file 1 [file animals-16-00311-s001.zip › animals-4056309-supplementary.pdf]

Table S1 Composition and nutrient levels of basal diets (air-dry basis) (%)

Ingredient	Content	Nutrition levels[2]	Content
Corn	60.50	DE(MJ/kg)	14.11
Fish meal	5.00	CP	20.21
Corn gluten meal	5.00	Ca	0.76
Soybean oil	1.00	AP	0.45
Soybean meal	24.00	Lys	1.70
Limestone	1.18	Met	0.49
$CaHPO_4$	1.30	Thr	1.05
L-Lys	0.60	Trp	0.28
Met	0.13		
Thr	0.17		
Trp	0.02		
Choline chloride	0.10		
NaCl	0.40		
Premix [1]	0.60		
Total	100.00		

[1] The premix provided the following per kilogram of the diet: VA 6 000 IU, VD_3 400 IU, VE 30 mg, VK_3 2 mg, VB_1 3.5mg, VB_2 5.5 mg, VB_6 3.5 mg, VB_{12} 25.0μg, biotin 0.05 mg, folic acid 0.3 mg, *D*-pAntothenic acid 20 mg, niacin 20 mg, choline chloride 500 mg, Fe (as ferrous sulfate) 110 mg, Zn (as zinc sulfate) 100 mg, Cu (as copper sulfate) 20 mg, Mn (as manganese sulfate) 40 mg, Se (as sodium selenite) 0.30 mg, I (as potassium iodide) 0.40 mg.

[2] Nutrient levels are calculated values.
